# Macro-Microscopical Sections of the Brain

**Published:** 1877-03

**Authors:** 


### Macro-Microscopical Sections of the Brain.

The brain, after removal of all enveloping tissues and vessels, is placed in common alcohol, where it remains about ten days, when absolute alcohol is substituted, to which tincture of iodine is added until the liquid is of the color of Maderia wine. It remains in this solution, which is frequently changed, also about ten days, when it is transferred to a saturated solution of bichromate of potash.

A human brain requires about two years before it is properly hardened, though the medulla, pons with the ganglia attached, and the brain of dogs, monkeys, and the fœtal brain may be hardened in three or four months.

To embed the brain in the cylinder of the microtome, it is to be dried and set in the desired position in a warm, flowing mass of stearine, fifteen parts, lard twelve parts, and white wax one part, melted together. On cooling, it is liable to shrink, and it is well to pour into the space thus contracted a solution of crude turpentine and wax melted together. The basin surrounding the cylinder is filled with water, and the sections are then cut under water.

The cut section is placed in water and allowed to remain some hours; it is then removed to a solution of carmine quite free from the odor of ammonia, and after twelve hours it is transferred to water slightly aciduluted with acetic acid. After remaining over night in this liquid, it is transferred to common alcohol and then to absolute alcohal, remaining about an hour in each. It is then removed to the glass, and, when the alcohol is evaporated, it is pencilled over with oil of cloves. When this is carefully done, so that there shall be no superfluous oil, Dammar lac is then poured over, and the upper glass adjusted. This should remain level one or two days, then, after removing the old superfluous Damar lac from the edges, a thin layer of melted wax is laid with a brush over the edges, and when cool, over this again, a preparation of asphalt and ether. Dr. Denny added that in his specimens, after the above preparation, he applied also a solution of caoutchouc in chloroform over the edges, and then covered both edges and sides with white wax. Oil of turpentine exposed to the air until it becomes thick may be used to render the specimen transparent instead of cloves.—*Dr. Denny, in Boston Med. and Surg. Journal.*

